# Prevention of rheumatic heart disease in New Zealand: High-dose subcutaneous benzathine penicillin is cost-saving compared with traditional intramuscular injections

**DOI:** 10.1016/j.ijregi.2025.100784

**Published:** 2025-10-06

**Authors:** William Leung, Michael G. Baker, Laurens Manning, Julie Bennett

**Affiliations:** 1 Department of Public Health, University of Otago, 43 Hanson Street, Wellington 6021, New Zealand; 2 Department of Medicine, University of Auckland, Private Bag 92019, Auckland 1142, New Zealand; 3 The Medical Research Institute of New Zealand, Private Bag 7902, Wellington 6242, New Zealand; 4 The Kids Research Institute Australia, Perth Children’s Hospital, 15 Hospital Avenue, Nedlands, Western Australia, 6009, Australia; 5 Medical School, University of Western Australia, 35 Stirling Highway, Crawley, Western Australia, 6009, Australia; 6 Department of Infectious Diseases, Fiona Stanley Hospital, 11 Robin Warren Drive, Murdoch, Western Australia, 6150, Australia

**Keywords:** Penicillin, Intramuscular, Subcutaneous, Rheumatic fever, Cost-saving

## Abstract

**Objectives:**

Acute rheumatic fever is a preventable condition that can lead to chronic illness and early death. Standard prevention with 4-weekly intramuscular (IM) benzathine penicillin G (BPG) injections for ≥10 years may be associated with poor adherence. High-dose 10-weekly subcutaneous penicillin injections (SCIP) may improve adherence by reducing injection frequency. Since 2025, SCIP has been included in the repository of standard operating procedures in lower North Island hospitals in New Zealand (NZ). We compare the costs of delivering a full course of secondary prophylaxis using SCIP vs IM BPG injections in NZ children with a first acute rheumatic fever presentation and no/mild carditis.

**Methods:**

Treatment administration costs and productivity losses from SCIP vs IM BPG were modeled using real-world survey data on time off school and work. Starting age of secondary prophylaxis, adherence, and administration site over the treatment period were varied. Costs are in 2024 NZ$, discounted at 3.5%.

**Results:**

SCIP reduced 12-month costs by nearly half to NZ$ 1629 for a 10-year-old child. Over a full course, SCIP consistently offered societal cost-savings, especially in younger children and higher adherence scenarios.

**Conclusions:**

Prevention using SCIP is cost-saving to NZ society. Increased adherence to SCIP may contribute to longer, healthier lives for those diagnosed with rheumatic fever.

## Highlights

• Subcutaneous injection of penicillin nearly halves 12-month treatment costs for 10-year-old children with rheumatic fever compared with intramuscular benzathine penicillin G.
• Cost-savings are greatest in younger children and when adherence is high.
• Subcutaneous injection of penicillin may improve adherence, reduce school absences, and prevent disease progression.
• Less frequent injections reduce nurse time, travel, and caregiver burden.

## Introduction

Acute rheumatic fever (ARF) is a preventable immune-mediated inflammatory condition that develops as a delayed response to Group A *Streptococcus* infections [1,2]. ARF and its complication, rheumatic heart disease (RHD), usually begin in childhood and can result in chronic illness, cardiac failure, stroke, and premature death [2]. Although the majority of global RHD burden occurs in low- and middle-income countries [3], Australia and New Zealand (NZ) have some of the highest reported ARF/RHD rates, disproportionately affecting Aboriginal, Māori, and Pasifika children [4].

For 70 years, the only proven way to prevent ARF progression has been benzathine penicillin G (BPG), administered via intramuscular (IM) injection every 4 weeks, usually for a minimum of 10 years. Good adherence is defined as receiving ≥80% of prescribed doses, with patients who adhere to <80% at a higher risk of developing RHD. The pain, anxiety, and frequency associated with IM BPG injections are often cited as barriers to adherence. To improve adherence, reduce treatment burden, and prevent disease progression, there is an urgent need to improve the delivery and formulation of long-acting penicillin. To address these challenges, recent phase I and phase IIa trials have explored high-dose subcutaneous injections of penicillin (SCIP), showing that a single injection can maintain therapeutic levels for at least 10 weeks [[5], [6], [7]]. These findings suggest SCIP may improve adherence and reduce burden, supporting its inclusion in standard operating procedures in lower North Island hospitals in NZ since May 2025. To explore the feasibility and potential value of SCIP as an alternative to standard care, this study compares the costs of delivering a full course of secondary prophylaxis using SCIP vs IM BPG injections in NZ children with a first ARF presentation and no/mild carditis.

## Methods

The costs of treatment administration and its resultant productivity losses for SCIP vs IM BPG were modeled in children diagnosed with ARF between 5 and 16 years of age. Treatment was until the patient was 21 years old or for 10 years, whichever was longer. For children aged <16 years, it was assumed nurses visited the child to administer SCIP or IM BPG, and, from age 16 years, the patient visited a medical center. For SCIP, each 10-week dose required 6-9 pre-filled 2.3-ml BPG glass syringes (Bicillin-LA®, Pfizer), depending on the patient’s weight. IM BPG was given as a single 2.3-ml BPG syringe every 4 weeks.

To estimate the average time off school or work due to post-injection pain, we surveyed patients residing in the Wellington region (n = 40) currently receiving SCIP treatment in our SCIP trial. From April to June 2025, the trial’s nurses contacted participants via text, telephone, or during appointments. A total of 22 (55%) participants responded. Their median age was 17 years (range: 11-46 years), and the median time since first ARF diagnosis was 7 years and 4 months.

Based on survey results, the model assumes that, on average, children receiving SCIP miss 3 hours of school due to post-injection pain vs 9 hours for IM BPG. Productivity losses (weighted by averaged Māori/Pasifika adult workforce participation) were estimated for caregivers of children aged 5-13 years because this age-group cannot be legally left home alone in NZ. For 14–17-year-old children, a day of missed school (weighted by the percentage of Māori/Pasifika children staying at school) [8] and missed work (for the 29% aged 16-17 years who have left school) were costed. From age 18 years, work productivity loss from treatment administration, travel, and post-injection pain was assumed, on average, to be 1.5 hours per SCIP dose and 2 hours per IM BPG dose (weighted by age-group specific workforce participation rates).

Costs are in 2024 NZ$, exclusive of goods and services tax, with a 3.5% discount rate for timing differences across years. As recommended by the NZ government, a societal perspective is adopted. Three modeled treatment adherence scenarios are explored: “perfect” adherence (SCIP five of five and IM BPG 13 of 13), “recommended” ≥80% adherence (SCIP four of five and IM BPG 11 of 13), and “lower” IM BPG adherence (10 of 13 when aged <16 years, and seven of 13 from age 16 years). These scenarios reflect a plausible range of adherence behaviors, with the ≥80% scenario aligned to NZ guidelines and the lower-adherence scenario reflecting real-world challenges reported in the literature [9,10].

Ethical approval for the SubCutaneous Injections of Penicillin trial was obtained from the NZ Health and Disability Ethics Committee (approval number 11094).

## Results

Table 1 shows the estimated 12-month costs for SCIP (NZ$ 1629) vs IM BPG (NZ$ 3132) for a 10-year-old child, the age when ARF is most commonly first diagnosed [4]. For children on SCIP, two-thirds of the total cost is from BPG, whereas for children on IM BPG, caregiver productivity loss from staying home to look after a child accounts for two-thirds of the total cost. The costs for nurse time and travel are lower for SCIP due to less frequent injections.

**Table 1:** Cost-analysis variables, with example of 12-month societal costs at 10 years of age.

Variable	Value^a^ (2024 NZ$)	Source	SCIP: age 10 years With 4 visits	IM: age 10 years With 11 visits
**Injection costs**				
BPG injection 900 mg (1.2 million units) in 2.3-ml syringe	$37.60	[11]	$1052.72	$413.57
Lidocaine injection 2%, 5-ml ampoule	$0.36	[11]	$1.44	$3.96
22 g canula (SCIP)	$6.96	[12]	$27.84	
Plaster to hold cannula in place (SCIP)	$0.91	[12]	$3.65	
District nurse time & mileage per visit to patients aged <16 years:				
- SCIP visit: 14 mins & 30 mins travel	$73.36	[12]	$293.43	
- IM visit: 6 mins & 30 mins travel	$59.63			$655.88
Visit to practice nurse for SCIP or IM when patients aged ≥16 years	$60.00			
**Productivity-loss–related**				
Māori/Pasifika adults labor force participation (caregiver)	69.4%	[13]		
Māori/Pasifika median hourly wage (caregiver)	$29.99	[13]	$249.58	$2059.01
Missed day of schooling (if age 14-17 years)	$55.56	[8]		
Median hourly wage (age 15-19 years)	$23.70	[13]		
Median hourly wage (age 20-24 years)	$27.00	[13]		
Māori/Pasifika staying on at school until age 17 years in 2023	70.8%	[13]		
Māori/Pasifika 15-19 years in employment, education or training in 2024	85.7%	[13]		
Māori/Pasifika 20-24 years in employment, education or training in 2024	76.9%	[13]		
**Example 12-month societal costs at age 10 (receiving ≥80% of visits)**			$1628.65	$3132.42

a All 2024 values, except “Māori/Pasifika staying on at school until age 17 years” (2023). BPG, benzathine penicillin G; IM, intramuscular; SCIP, SCIP-II Phase-II trial of subcutaneous injections of BPG.

Over a full course of secondary prophylaxis, societal costs are typically lower for SCIP than for IM BPG injection (Table 2). For patients receiving the recommended ≥80% of treatment visits, SCIP consistently provides cost-savings for children who first begin secondary prophylaxis age 5-14 years, with the greatest cost savings at NZ$ 12,958 for 5-year-old children. In the lower-adherence scenario, SCIP result in smaller savings for children who first begin secondary prophylaxis at age 5-11 years.

**Table 2:** Discounted societal costs (2024 NZ$, 3.5%) of a full course (the longer of until age 21 years or duration of 10 years) of secondary prophylaxis administration in children presenting with acute rheumatic fever and no/mild carditis.

Treatment adherence		*Perfect (100%)*			*Recommended (≥80%)*			*Lower than recommended for IM BPG*		
Starting age (years)	SCIP dosing by age^a^	SCIP (5 pa)	IM (13 pa)	Savings with SCIP	SCIP (4 pa)	IM (11 pa)	Savings with SCIP	SCIP (4 pa)	IM (<16 years = 10 pa; ≥16 years = 7 pa)	Savings with SCIP
5	6	24,633	38,626	13,994	19,695	32,653	12,958	19,695	28,216	8521
6	6	23,582	36,147	12,564	18,855	30,554	11,699	18,855	23,257	7402
7	6	22,495	33,580	11,085	17,985	28,381	10,397	17,985	24,228	6244
8	6.5	21,370	30,924	9554	17,084	26,133	9048	17,084	22,129	5045
9	6.5	20,108	28,175	8067	16,074	23,805	7731	16,074	19,956	3882
10	7	18,802	25,330	6527	15,029	21,396	6367	15,029	17,707	2678
11	7	17,353	22,385	5031	13,869	18,903	5034	13,869	15,380	1510
12	7	17,459	20,667	3207	13,954	17,448	3494	13,954	13,687	–267
13	7	17,569	18,889	1319	14,041	15,942	1901	14,041	11,935	–2106
14	7.5	17,683	17,049	–635	14,132	14,384	252	14,132	10,122	–4010
15	7.5	17.947	16,627	–1319	14,346	14,039	–307	14,346	9406	–4940
16	8	18,235	16,247	–1988	14,581	13,730	–851	14,581	8695	–5886

a Estimated average number of pre-filled 2.3-ml BPG glass syringes (Bicillin-LA, Pfizer) per SCIP dose by age. Increasing to 8.5 pre-filled BPG syringes required from age 18 years and to nine syringes from age 20 years and older. BPG, benzathine penicillin G; IM, intramuscular, pa per annum; SCIP, subcutaneous injections of BPG.

## Discussion

This preliminary analysis shows that a full course of secondary prophylaxis with SCIP is cost-saving compared with traditional IM BPG for NZ children first diagnosed with ARF before the age of 15 years. Societal cost savings with SCIP increase the younger the children are when they begin secondary prophylaxis. Because children receiving IM BPG take more time off school due to post-injection pain compared with SCIP, caregivers incur more productivity losses because children aged <14 years cannot legally be left home alone in NZ.

Although some caregivers may work from home when children miss school due to post-injection pain, this is less likely given the socioeconomic gradient of rheumatic fever. Our time off estimates, although based on a small survey, align with the experiences of five nurses providing IM BPG. Some costs, such as nurse travel, may be over-estimated when multiple children are treated in a single visit.

Cost-savings could be under-estimated because lower IM BPG adherence can increase disease progression, healthcare utilization, productivity losses, and preventable mortality. SCIP has been shown to be preferred due to less pain and longer dosing intervals, which may improve adherence [12]. A lifetime Markov model is being developed to assess the long-term cost-effectiveness and quality-of-life impacts of SCIP on patients with RHD.

The generalizability of our findings is limited to high-income countries, where it is illegal to leave a child (before their teenage years) home alone. Societal cost savings will be reduced if a child can be left home alone at a younger age. In jurisdictions without such laws in place, parents can still be prosecuted if they leave a child unsupervised in a manner likely to cause unnecessary suffering or injury to health. Therefore, it is likely that caregivers will still be required, particularly, for younger children (who have to stay home due to post-injection pain), and that treatment with SCIP will result in annual societal cost savings.

## Conclusion

In addition to being cost-saving to NZ society, increased adherence to SCIP may contribute to longer, healthier lives for those diagnosed with rheumatic fever. Due to potential global shortages of Bicillin-LA, future research should explore the possibility of using powdered formulations.

## Declaration of competing interest

The authors have no competing interests to declare.
